# A modeling study on utilizing low temperature sprayed In_2_S_3_ as the buffer layer of CuBaSn(S, Se) solar cells

**DOI:** 10.1038/s41598-021-99012-6

**Published:** 2021-10-08

**Authors:** Maryam Hashemi, Mehran Minbashi, Seyed Mohammad Bagher Ghorashi, Arash Ghobadi

**Affiliations:** 1 Department of Laser and Photonics, University of Kashan, P.O. Box 873175-3153, Kashan, Iran; 2 Department of Physics, Tarbiat Modares University, P.O. Box 14115-175, Tehran, Iran; 3 Department of Physics and Astronomy, University of Missouri, Columbia, MO 65211 USA

**Keywords:** Chemistry, Energy science and technology, Materials science, Nanoscience and technology, Optics and photonics, Physics

## Abstract

This study represents the investigation of In_2_S_3_ thin films as an electron transport layer in the CuBaSn(S, Se)-CBT(S, Se) solar cells, which have been deposited using the Chemical Spray Pyrolysis method. For studying the electrical properties of films such as conduction and valence band, carrier densities, Fermi level, flat band potential, and semiconductor type, the Mott–Schottky analysis has been used. UV–VIS, XRD, and FESEM have been applied to investigate the optical properties of the layers and the layer’s morphologies. The experimental CBT(S, Se) solar cell has been simulated and validated as the next step. After that, the In_2_S_3_ layer has been used as the electron transport layer. The results represent that the In_2_S_3_ layer is a suitable substitution for toxic CdS. Finally, the In_2_S_3_ properties are varied in reasonable ranges, which means different electron transport layers are screened.

## Introduction

Urbanization and rapid growth in industrialization extensively bring significant increments in environmental pollution and global warming, which are vital issues alongside the energy crisis that oblige scientists to seek a suitable alternative energy source to rescue the earth and the environment. Thin-film photovoltaic (PV) solar cell technology has grasped global attention among researchers due to its outstanding promises for renewable energy resources and substitution with fossil fuels to meet human energy needs^1–12^. Global efforts have been devoted to select (1) non-toxic, (2) air-stabile, and (3) environmentally friendly earth-abundant compositions to manufacture highly efficient thin-film solar cells^13–17^. Chalcogenide compounds are well-known semiconducting materials, which can gain considerable attention among scientists due to their narrow bandgap and capability to use PV devices and solar cells^18–21^.

Among chalcogenide compounds, chalcopyrite Cu(In,Ga)Se_2_-(CIGS) and CdTe have demonstrated an essential platform for clean renewable energy generation with respective efficiency of 23.35%^22^, and 22.1%^23,24^, respectively. Supply limitations for In, Ga, and Te and the toxicity of Cadmium (Cd) are the biggest deficiencies for large-scale production of these PV devices. Moreover, kesterite (KS) alloys Cu_2_ZnSnS_4_-(CZTS), Cu_2_ZnSnSe_4_-(CZTSe), and Cu_2_ZnSn(S,Se)_4_-(CZTSSe) are considered as potential replacements for the chalcopyrite CIGS and CdTe absorber materials since they do not contain toxic and scarce elements. Cationic disorder and the existence of intrinsic defects form unwanted defects and defect complexes, which directly affect the carrier concentration, electrical conductivity, elemental non-stoichiometry, and the cell power conversion energy (PCE)^19,25–28^.

Cu_2_BaSnS_x_Se_4−x_ (CBTSSe) has been investigated as a thin-film absorber with similar optoelectronic features to target failures of the other chalcogenide semiconductors. Eco-friendly and non-toxic material with a direct bandgap (~ 2 eV), high absorption coefficient (> 10^4^ cm^−1^), *p* type conductivity, and appropriate defect properties could gain a prominent position in the world of chalcogen-based PV devices, which motivate scientists to put more effort to investigate them^14,29–32^.

Despite the major methods done to improve the overall performance of solar cells, utilizing a suitable n-type electron transport layer creates a way to enhance the electrical characteristics of the cell, especially the open-circuit voltage (V_OC_), short-circuit current density (J_sc_), and fill factor (FF) (%) as well as the cell efficiency. The band alignment at the interface of the absorber/buffer drastically affects cell parameters. It is worth mentioning that an electron transport layer with an optimal conduction band offset (CBO) reduces the V_OC_ deficit, which directly affects the device performance^33–35^.

Indium sulfide (In_2_S_3_) stands out as a promising candidate among the other semiconducting materials to use as an electron transport layer in solar cell fabrication. This n-type material has attracted considerable attention among scientists due to its wide direct bandgap (2.0–2.8 eV), non-toxic components, chemical stability, high transparency in the visible region, and photoconductive nature^36–39^.

Among all the other simulation software, the solar cell capacitance simulator (SCAPS-1D) has been employed in this work to calculate photovoltaic parameters, such as open-circuit voltage (V_OC_), short-circuit current density (J_sc_), and efficiency (η) under standard illumination (AM 1.5 solar radiation/the power density of 100 mW/cm^2^ is used as the source of illumination)^40–43^. The calculations of this software are based on the Poisson and the Continuity equations for electrons and holes. The numerical study is carried out to represent an accurate work with optimum model parameters and investigate the influence of physical parameters on the device’s performance.

In this work, we used the Chemical Spray Pyrolysis method to deposit In_2_S_3_ thin films as the electron transport layer and study their morphological, structural, optical, and electrochemical properties. This way not only enabled the low-temperature deposition of In_2_S_3_ at 250 °C but also supplied the ability to use the non-toxic material of the In_2_S_3_ at the Cu_2_BaSnS_x_Se_4−x_ (CBTSSe) solar cells. In order to get more insight on the influence of In_2_S_3_ thin films on device performance, theoretical current density–voltage characteristics under irradiation are investigated. Device modeling and numerical simulations of CBTSSe/In_2_S_3_ solar cells are achieved to examine the impacts of the valence band offset, conduction band offset, donor density, electron mobility, the thickness of buffer layer, effective density of the conduction band states, absorption coefficient constant, interface density affect a proposed structure’s performance. For this purpose, numerical simulations of electrical responses of the solar cell device are made using the solar cell capacitance simulator (SCAPS-1D) program (version 3.3.05). The numerical simulation results propose an optimal geometrical structure for the CBTSSe/In_2_S_3_ solar cells.

## Material and methods

### Material

The In_2_S_3_ molecular-based precursor solution was prepared using a mixture of chloride metal salt and thiourea (TU) dissolved in water and ethanol (Merck 98%) with a ratio of 1 (ethanol):3 (water) to study the effect of low-temperature deposition on electron transport layer properties. Then, Indium (III) chloride (InCl_3_, STREM CHEMICALS, 99.99%), and thiourea (ACROS,99%) was used as indium and sulfur source. The molar ratio of In:S was 1:4. All chemicals were used without further purification.

### Experimental method

**Figure 1:**
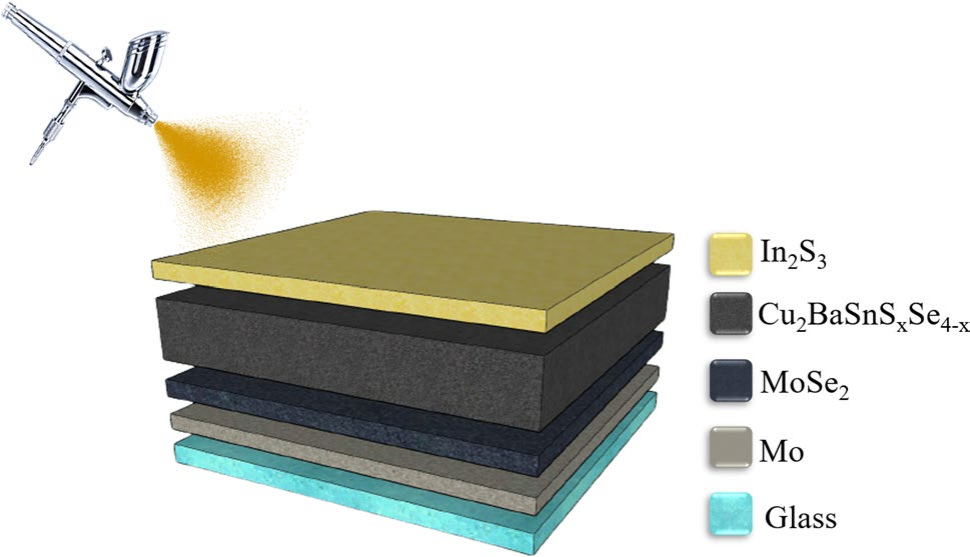
The scheme of the spray pyrolysis set-up.

The investigation of the effect of the low-temperature electron transport layer by means of In_2_S_3_ molecular-based precursor solution has been explained above. For more explanation, with 100% consideration, 0.1 M of thiourea concentration and 25 mM of indium concentration were kept constant for all experiments. The samples were located on a hot plate in the air with a surface temperature of 250 °C to deposit In_2_S_3_ layers. It must be stressed that the surface temperature should be constant over the sample. The rate of deposition 4 ml/min, 17 cm height (distance between hot plate and nozzle), and 250 °C deposition temperature are the optimum conditions for In_2_S_3_ deposition^44^. The carrier gas was the air with a constant rate of 3 L/min. The total volume of the solution sprayed and the minimum size of the samples were 2 ml and 1.1 in. × 1.1 in., respectively. As one can see, Fig. 1 represents the scheme of the spray pyrolysis set-up.

## Device analysis

Philips XL30 system has been employed for FESEM investigation of the layer morphologies. Moreover, X-ray diffraction (X’Pert Pro MPD, PANalytical) was the method to analyze the crystal structural properties. The optical properties of the deposited layers were figured out by calculating the transmittance spectra using UV–Vis spectroscopy (Lamda 25, Perkin Elmer). The Mott-Schottky analysis was utilized in a three-electrode configuration, using a quartz cell and 0.5 M Na_2_SO_4_ solution (pH 6.0) using an IRASOL EIS-26H system. The working reference and counter electrodes were In_2_S_3_/FTO, Ag/AgCl (3 mol/kg KCl), and Pt. The signal frequency was 1 kHz, and the bias voltage was scanned from − 0.8 to 0.3 V, with 50 mV/s speed (peak-to-peak) in the environmental condition.

## Results and discussion

### Structural and optical properties

**Figure 2:**
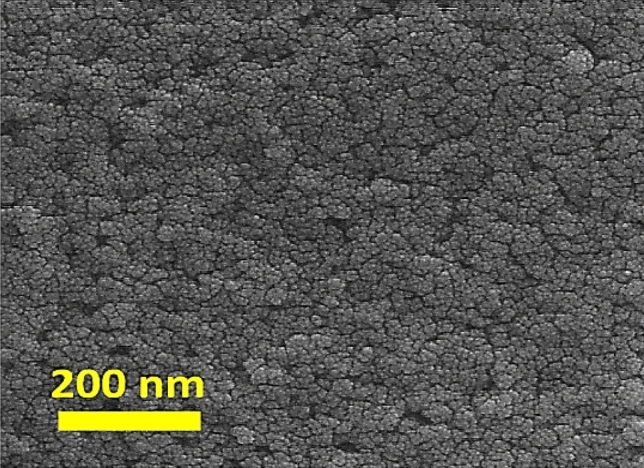
FESEM images of In_2_S_3_ layers prepared by spray method at 250 ℃.

Figure 2 demonstrates the side view field emission scanning electron microscope (FESEM) of the In_2_S_3_ layer. The In_2_S_3_ layers prepared have a cauliflower, compact, homogenous, and crack-free structure. A compact In_2_S_3_ electron transport layer can improve the interfacial contact with CBT (S, Se) layers and boost cell performance. The ideal connection between the In_2_S_3_ and CBT (S, Se) is expected to provide a perfect pathway for photo-generated carriers^45^. The usual transparent conductive electrode with a compact blocking structure used as the front contact in CBT (S, Se) solar cells is the indium tin oxide (ITO). The primary responsibility of this thin layer is as a front electrode is reducing series resistance^46–48^.

**Figure 3:**
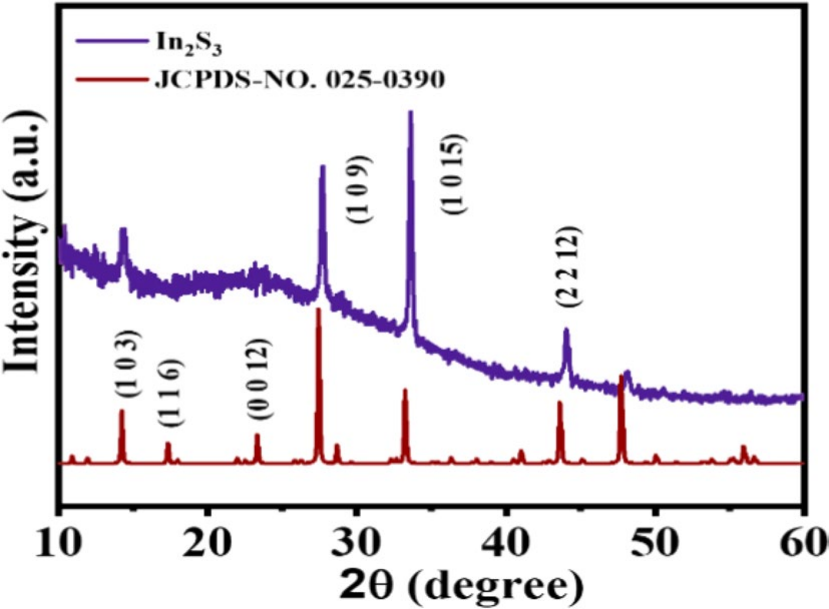
Comparison of XRD diffraction spectra of In_2_S_3_ layers with the standard card.

According to Fig. 3, the XRD spectra helps to investigate phase compounds and the crystalline structure of the layer. Figure 3a depicts the spectral resolution of the In_2_S_3_ layer in comparison to the (JCPDS.NO. 25-0390) standard card. Peak diffraction (1 0 15) is characteristic of the formation of the tetragonal structure, which contrasts with the cubic structure. Notably, the peak diffraction (1 0 15) of the crystallization characteristic tetragonal sequentially because of the diffraction peaks of the two-crystal cubic and tetragonal structures overlapped^44^.

Additionally, none of the oxide compounds peaks were found in the XRD pattern, which means that these compounds are not present in the accuracy of XRD analysis detection. Some works reported that sulfur produces exhaustion of oxygen in the proximity of the film that prevents the formation of indium oxide^49^.

**Figure 4:**
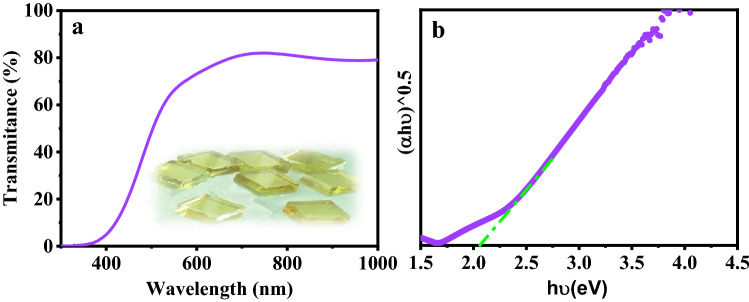
(**a**) Optical transmittance spectra and (**b**) indirect bandgap of In_2_S_3_ layers.

The UV–Vis study was carried out to characterize the total optical transmittance to define the optical bandgap of the In_2_S_3_ layers. As can be seen, Fig. 4a illustrated the transition spectrum and indirect bandgap energy of the In_2_S_3_ layer prepared at 250° C. The prepared In_2_S_3_ layer revealed fine transparency in the spectral range of 300 1100 nm. For wavelengths smaller than the energy gap, the layer’s transparency is about 90% in the visible and near-infrared region of the spectrum. The optical absorption coefficient (α), electron transfer, is related to the energy gap and is defined in terms of (α ≥ 10^4^ cm^−1^) as (Eq. 1):

1 $$\alpha \cong \frac{{A\left( {hv - E_{g} } \right)^{n} }}{hv}$$

where A is a constant that depends on the transition probability, E_g_ shows the energy gap, $hv$ is the incident photon energy, and $n$ is a parameter that describes the electron transfer between the conduction and valence bands. $n = 2$ for an indirect electron transfer. Thus, according to Fig. 4b, the E_g_ value for indirect transition is determined by extrapolating the linear part of the plot ($\alpha hv$)^0.5^ versus ($hv$) in the abscissa (axis x), which depicts an indirect optical transition. The E_g_ value of the In_2_S_3_ layer deposited was 2.12 eV (indirect transitions).

### Electrochemical properties

**Figure 5:**
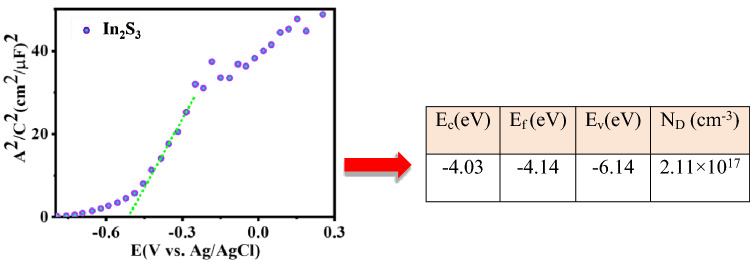
The Mott–Schottky (M–S) analysis curve of In_2_S_3_ layers measured in 0.5 M Na_2_SO_4_.

All semiconducting properties of materials, such as the free-carrier concentration, flat-band potential, energy levels, and conductivity type, are critical components that lead to better device performance. These properties can be determined from a Mott–Schottky (M–S) plot consisting of the space-charge-layer capacitance (C_SC_) inverse square versus the bias potential^50^. It is well defined that the slope of such plot is used to determine the semiconductor doping density *N*_D,_ and extrapolation of intercept may be used to compare the flat band potential *V*_fb_, Fig. 5, using the well-known Mott–Schottky relation (Eq. 2):

2 $$\frac{1}{{C_{SC}^{2} }} = \frac{2}{{\varepsilon_{0} \varepsilon_{r } eN_{D} }}\left( {V - V_{fb} - \frac{KT}{e} } \right)$$

which *N*_*D*_, *e*, *ε*_*0*_, *ε*_*r*_, *V*_*fb,*_ and *V* are carrier concentration, electron charge, vacuum permittivity, the dielectric constant of semiconductor, flat band potential, and applied potential, respectively. *T* is the temperature of the operation (300 K), *K* is the Boltzmann’s constant (1.38 × 10^−23^ J/K), and *C*_*SC*_ is the space charge capacitance. In this work dielectric constant for In_2_S_3_ films is equal to 8.5. As Mott–Schottky plot depicts (Fig. 5), the positive slope of the curves indicates that films all have an n-type conductivity^51^.

To understand precisely, the carrier density (*N*_*D*_) has been calculated alongside the calculation of the In_2_S_3_ carrier transport. From the Mott-Schottky (M–S) curve slope, the shallow donor density derived 2.11 × 10^17^ cm^−3^ for In_2_S_3_. The flat band potential (*V*_*fb*_) is the other parameter, which has been investigated. The redox potentials are conveniently expressed in the band edges by flat band potential (*V*_*fb*_). Actually, *V*_*fb*_ is the needed potential for applying to the semiconductor to reduce the band bending to zero. The flat band potential is obtained as − 0.74 V (relative to Ag/AgCl) for the In_2_S_3_ layer. The different *V*_*fb*_ values emerge as a result of conductivity differences among the other crystallographic planes.

## Numerical study

As mentioned above, SCAPS-1D software was the package, which has been used for the simulated part of the current work. The numerical model of this work has been validated with the experimental data to define instructions for better illustrating the cell PCE improvement. The validation results are available in the supporting information file.

### Investigation of In_2_S_3_ as the electron transport layer

The effect of substituting a uniform, thin, and continuous interfacial In_2_S_3_ layer as the electron transport layer with toxic CdS has been investigated in the present study, which is based on examining the following parameters.

#### Examination of the valence band offset (VBO)

It should be stated that the CBT (S, Se) solar cell characteristics define in accordance with the buffer layer valence band offset (VBO) and conduction band offset (CBO).

3 $$VBO = \left( {\chi_{{CBT \left( {S,Se} \right)}} + E_{{g_{{CBT \left( {S,Se} \right)}} }} } \right) - \left( {\chi_{buffer} + E_{{g_{buffer} }} } \right) = 1.696 - E_{{g_{buffer} }}$$

**Figure 6:**
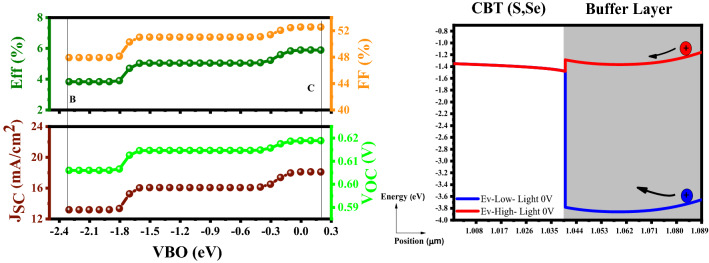
Variation of the solar cell parameters of the CBT (S, Se) solar cells with different VBO of the In_2_S_3_ layer.

According to Fig. 6, increment in VBO leads to PCE enhancement because hole particles at point C (E_V_-High) can easily flow from the buffer to the absorber without spending additional energy. An increment in open-circuit voltage (V_OC_), short-circuit current density (J_sc_), and Fill Factor (%), as well as the PCE, would be the outcome. On the other hand, the blue hole particle at point B (E_V_-Low) represents trapped in the well passing needs a considerable amount of energy, and it is impossible for a hole to gain this high amount of energy so, the consequence would be a reduction in parameters and PCE^52,53^. Functional parameters for two selected points are gathered in Table 1.

**Table 1:** Simulation characteristics of the CBT (S, Se) solar cells by utilizing the In_2_S_3_ electron transport layer as a function of VBO.

Point	VBO	Value	Efficiency (%)	FF (%)	JSC (mA/cm^2^)	VOC (V)
B	− 2.304	Low	3.83	47.93	13.19	0.60
C	0.196	High	5.89	52.55	18.10	0.619

#### Examination of the conduction band offset (CBO)

The electron affinity (χ) defines the conduction band offset (CBO) according to the following equation (Eq. 4):

4 $$CBO = \chi_{buffer} - \chi_{{CBT \left( {S,Se} \right)}}$$

The electron affinity of the electron transport layer should be more than the absorber’s because, in this case, CBO becomes higher. As a result, the electron can easily cross the electron transport layer to enhance the open-circuit voltage (V_OC_) and short-circuit current density (J_sc_). Higher V_OC_ and J_SC_ lead to lower recombination of photo-generated carriers and thus enhances the PCE.

**Figure 7:**
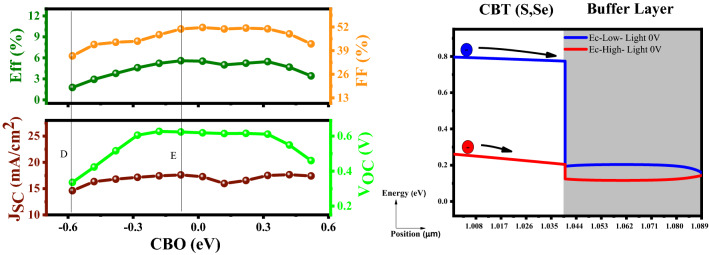
Variation of the solar cell parameters of the CBT (S, Se) solar cells with different CBO of the In_2_S_3_ layer.

As one can see in Fig. 7, CBO enhancement brings better performance of the device. Electron particles in the absorber in E_c_-High mode (point E) can simply move to the electron transport layer due to the steeper slope toward the buffer layer. This drastically increases the open-circuit voltage (V_OC_), short-circuit current density (J_sc_) as well as the cell efficiency. On the other side, there is no barrier for the electron to flow to the electron transport layer E_c_-Low mode (point D), but the movement velocity is lower than the previous mode^54^. Table 2 represents the functional parameters for two selected points.

**Table 2:** Simulation characteristics of the CBT (S, Se) solar cells by utilizing the In_2_S_3_ electron transport layer as a function of CBO.

Point	CBO	Value	Efficiency (%)	FF (%)	JSC (mA/cm^2^)	VOC (V)
D	− 0.58	Low	1.77	36.09	14.60	0.335
E	− 0.08	High	5.58	50.90	17.60	0.623

#### Examination of the donor density (N_D_)

**Figure 8:**
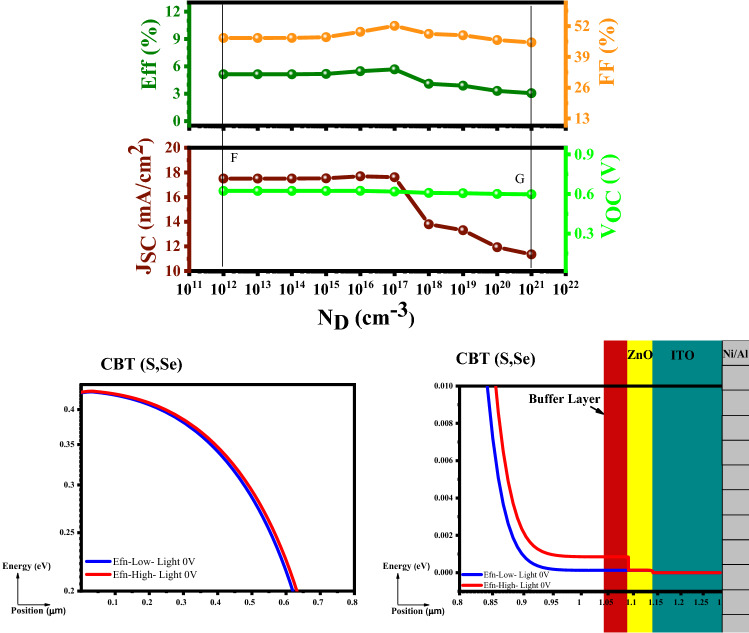
Variation of the solar cell parameters of the CBT (S, Se) solar cells with different N_D_ of In_2_S_3_ layer.

Based on the Fermi level (E_fn_) definition, its position fixes the charge carrier concentration, and higher-level leads to higher short-circuit current density (J_sc_) as well as the efficiency. As can be seen in Fig. 8, E_fn_-high (Red line) depicts a higher Fermi level, which gradually decreases by changing the position. According to the E_fn_-high, the cell records its highest J_sc_ and PCE at N_D_ = 10^12^ cm^−3^. It should be stated that from N_D_ = 10^12^ to 10^17^ cm^−3^, all the PV parameters show a constant value, but after N_D_ = 10^17^ cm^−3^, the open-circuit voltage (V_OC_) remains constant, and Fill Factor (FF) changes is not so effectual. The only drastic change is in short-circuit current density (J_sc_), which directly affects the PCE.

#### Examination of the electron mobility (µ_e_)

**Figure 9:**
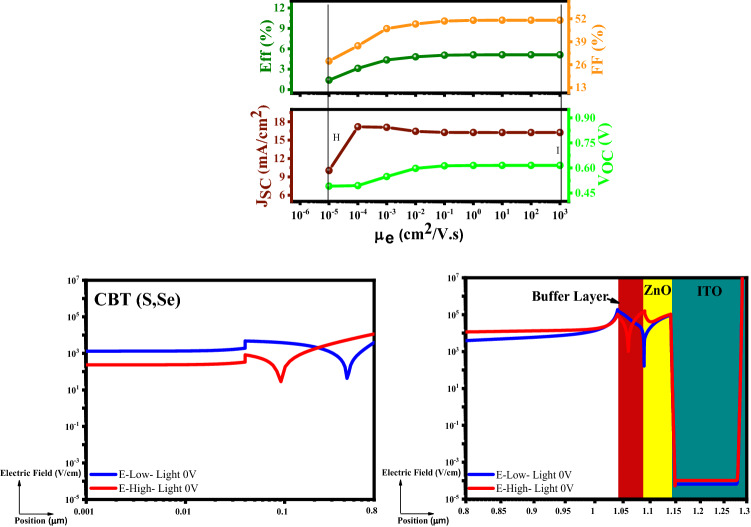
Variation of the solar cell parameters of the CBT (S, Se) solar cells with different μ_e_ of In_2_S_3_ layer.

Investigation of the electron mobility (µ_e_) is the next step (Fig. 9), which has been done in the range of 10^−6^–10^3^ cm^2^/V s. As a critical point, more electrons respond to the electric field rather than the holes due to the electron mobility increment (µ_e_). The increment of electron mobility (µe) helps the buffer layer catch more electrons from the conduction band. Therefore, the effect of the majority carrier denied, which increases the recombination at the back contact. Consequently, a better flow of electrons between the buffer/absorber interface causes an increment in the PCE. This could be sensible based on the following electric field graphs and integrations (electrostatic potentials); equation (Eqs. 5 and 6):

5 $$E{ - }Low \left( {Blue\;Line} \right) \to \left| \varphi \right| = \mathop \smallint \limits_{0}^{1.29 um} E.dl = 1.49 V$$

6 $$E{ - }High \left( {Red\;Line} \right) \to \left| \varphi \right| = \mathop \smallint \limits_{0}^{1.29 um} E.dl = 1.96 V$$

#### Examination of the thickness (μm)

**Figure 10:**
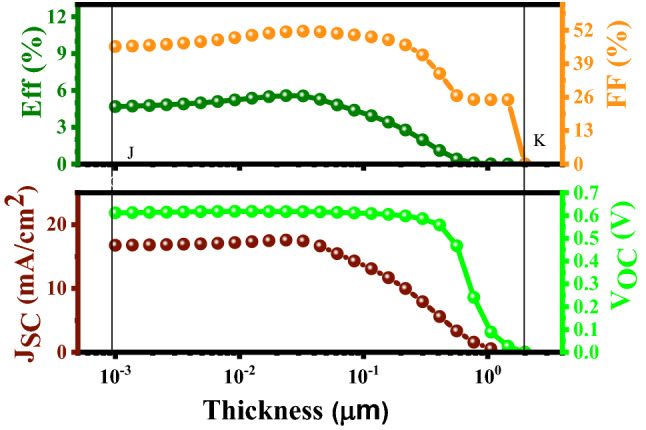
Variation of the solar cell parameters of the CBT (S, Se) solar cells with different thicknesses of the In_2_S_3_ layer.

As can be seen in Fig. 10, in the range of 10^−3^–10^−1^ μm (optimum depletion width), the cell parameters remain constant, as well as the PCE. By increasing the buffer thickness (reducing the depletion width), photon aggregation happens, and this significantly increases the recombination rate, which is detrimental for the cell performance because the short-circuit current density (J_sc_) goes to zero; subsequently, all the PV parameters become zero.

#### Examination of the effective density of the conduction band states (N_C_)

**Figure 11:**
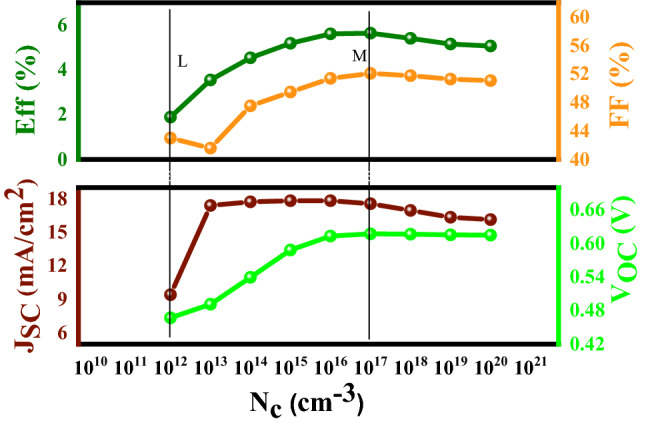
Variation of the solar cell parameters of the CBT (S, Se) solar cells with different N_C_ (cm^−3^) of the In_2_S_3_ layer.

The CBT (S, Se) is a p-type semiconductor, and according to its definition, for a p-type semiconductor, holes are the major charge carriers. Moreover, for a p-type semiconductor, the Fermi level (E_f_) position locates very close to the top of the valence band. According to Fig. 11, the effective density of the conduction band states (N_C_) can influence the cell performance in the range of 10^12^–10^17^ cm^−3^, but it has a negligible effect on cell parameters after a certain value (10^17^ cm^−3^).

#### Examination of the absorption coefficient constant (α)

**Figure 12:**
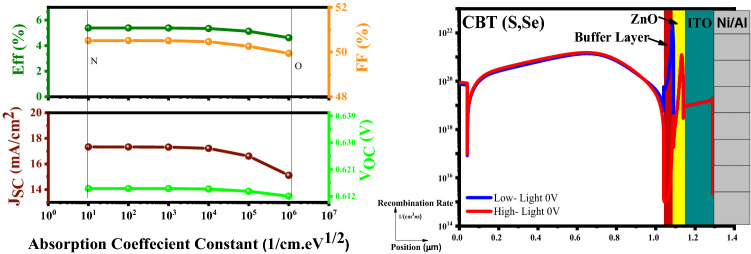
Variation of the solar cell parameters of the CBT (S, Se) solar cells with different the Absorption Coefficient Constant (1/cm eV^1/2^) of In_2_S_3_ layer.

Figure 12 depicts that the recombination rate increases due to the absorption increment, and as can be seen, the increment of the absorption deteriorated the cell performance.

#### Examination of the interface density (N_t_)

**Figure 13:**
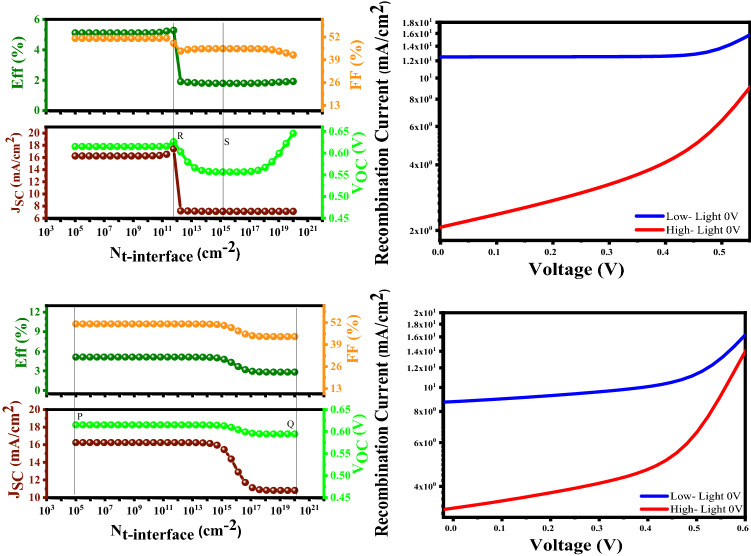
Variation of the solar cell parameters of the CBT (S, Se) solar cells with different N_t-interface_ (cm^−2^) of the In_2_S_3_ layer.

On the report of Fig. 13, higher N_t-interface_ records higher recombination current, and it would be detrimental for the cell parameters and dramatically reduces the device performance.

## Conclusion

This paper is a device modeling study to evaluate In_2_S_3_ thin films as a possible electron transport layer in CBT(S, Se) solar cells. We deposited In_2_S_3_ layers by low-temperature spray pyrolysis and characterized the films to obtain valid input values for modeling the device. The physical and electrical properties of In_2_S_3_ layers have been investigated. There is a good correlation between the results obtained from different characterization techniques. In_2_S_3_ thin films obtained revealed good transparency in the spectral range 300–1100 nm. The value of the indirect bandgap is measured as 2.11 eV. In_2_S_3_ films show a tetragonal ordering structure of β-In_2_S_3_. The FESEM analysis confirms that films have a homogenous, crack-free dense microstructure that covers the entire substrates perfectly. In addition, all the films have an n-type conductivity. The modeling results show that In_2_S_3_ is a promising material with electronic properties similar to CdS. In_2_S_3_ is an attractive material, and we believe that this material has a high potential for being optimized as an n-type buffer layer for CBT (S, Se) solar cells, as well as other types of solar cells. In the proposed optimized structure, it can be found that good buffer layer candidates for CBT (S, Se) solar cells.

## Supplementary Information

Supplementary Information.
