# Supplementary material for: A modeling study on utilizing low temperature sprayed In2S3 as the buffer layer of CuBaSn(S, Se) solar cells
Source: Sci Rep. 2021 Oct 8;11:20038. doi: 10.1038/s41598-021-99012-6 (PMC8501044; doi:10.1038/s41598-021-99012-6)
Supplement: Supplementary file 1 — Supplementary Information. [file 41598_2021_99012_MOESM1_ESM.pdf]

# ***Supporting Information***

# A modeling study on utilizing low temperature sprayed $In_2S_3$ as the buffer layer of CuBaSn (S, Se) solar cells

Maryam Hashemi[a], Mehran Minbashi[b*], Seyed Mohammad Bagher Ghorashi[a*], Arash Ghobadi[c]

[a]University of Kashan, Department of Laser and Photonics, P.O. Box 873175-3153, Kashan, Iran

[b]Department of Physics, Tarbiat Modares University, P.O. Box 14115-175, Tehran, Iran

[c]Department of Physics, Semnan University, P.O. Box: 35195-363, Semnan, Iran

[1] mehran.minbashi@modares.ac.ir ; mehran.minbashi@gmail.com
[2] mghorashi@kashanu.ac.ir

## 1. Validation

By considering the Shockley-Read-Hall (SRH) recombination statistics, and simultaneous basic semiconductor equations including the Poisson, Continuity, Drift, and Diffusion equations for electrons and holes, SCAPS-1D calculates PV parameters. Based on the Shockley-Read-Hall (SRH) recombination statistics (Eqs. S1-S5) [1–6]:

$$\frac{d^2}{dx^2}\psi(x) = \frac{e}{\varepsilon_0 \varepsilon_r}(p(x) - n(x) + N_D - N_A + \rho_P - \rho_n) \qquad (S1)$$

Where $\psi$ is electrostatic potential, e is electrical charge, $\varepsilon_r$ and $\varepsilon_0$ are the relative and the vacuum permittivity, p and n are hole and electron concentrations, $N_D$ and $N_A$ are charge impurities of

donor and acceptor type, $\rho_p$ and $\rho_n$ are holes and electrons distribution, respectively. The continuity equations for electrons and holes are:

$$\frac{dJ_n}{dx} = G - R \tag{S2}$$

$$\frac{dJ_p}{dx} = G - R \tag{S3}$$

Where $J_n$ and $J_p$ are electron and hole current densities, R is the recombination rate, and G is the generation rate. Carrier transport in semiconductors occurs by drift and diffusion and can be defined by the equations (S4) and (S5):

$$J_n = D_n \frac{dn}{dx} + \mu_n n \frac{d\phi}{dx} \tag{S4}$$

$$J_p = D_p \frac{dp}{dx} + \mu_p p \frac{d\phi}{dx} \tag{S5}$$

The present simulation is based on the experimental data reported in Ref.[7] for CBT (S, Se) thin-film solar cell with a record PCE of 5.2% In Ref.[7], the authors are of the opinion that among metal chalcogenides, using air-annealed $Cu_2BaSnS_{4-x}Se_x$ with x=3 records more than 2.5 times enhancement in cell performance. According to their astounding modification: annealing the $Cu_2BaSnS_{4-x}Se_x$ (x=3) film in the air at 200°C for 3 min, the device recorded $V_{OC}$= 611 mV, $J_{sc}$= 17.4 mA/cm$^2$, FF= 48.89 %, and PCE= 5.2(1) % for a total area of 0.425 cm$^2$, respectively [7,8].

The comparison between the simulation results and the experimental data demonstrates the precision of the simulated results. The parameter values used in the simulation has been collected in table S1.

Table S1: Parameters set for the simulation of CBT (S, Se) solar cell [9–12]

| **Parameters and Units** | **ITO** | **ZnO(i)** | **$In_2S_3$** | **CdS** | **CBT (S, Se)** |
|---|---|---|---|---|---|
| Thickness (nm) | 150 | 50 | 50 | 50 | 1000 |
| Electron affinity (eV) | 4.80 | 4.40 | 4.03 | 4.20 | 4.22 (Fitting) |
| Bandgap (eV) | 3.65 | 3.3 | 2.06 | 2.40 | 1.506 |
| Dielectric permittivity (relative) | 8.9 | 9.0 | 6.50 | 10.0 | 5.5 |
| CB effective density of states ($cm^{-3}$) | $5.2\times10^{18}$ | $1.0\times10^{18}$ | $1.8\times10^{19}$ | $1.0\times10^{18}$ | $1.76\times10^{18}$ |
| VB effective density of states ($cm^{-3}$) | $1.0\times10^{18}$ | $1.0\times10^{19}$ | $4.0\times10^{13}$ | $1.0\times10^{19}$ | $3.42\times10^{19}$ |
| Electron mobility ($cm^2/Vs$) | 10 | 100 | 400 | 100 | 5(Fitting) |
| Hole mobility ($cm^2/Vs$) | 10 | 25 | 210 | 25 | 1.5 |
| Shallow uniform donor density $N_D$ ($cm^{-3}$) | $1.0\times10^{20}$ | $1.0\times10^{17}$ | $2.11\times10^{17}$ | $1.0\times10^{18}$ | 0 |
| Shallow uniform acceptor density $N_A$ ($cm^{-3}$) | 0 | 0 | 0 | 0 | $5.0\times10^{14}$ |
| Absorption constant A (1/cm eV $^{(1/2)}$) | Data file (SCAPS Value) | Data file (SCAPS Value) | Data file (SCAPS Value) | Data file (SCAPS Value) | $5.1\times10^{4}$ |
| Absorption constant B (eV $^{(1/2)}$/cm) | | | | | 0(Fitting) |
| Electron thermal velocity (cm/s) | $1.0\times10^{7}$ | $1.0\times10^{7}$ | $1.0\times10^{7}$ | $1.0\times10^{7}$ | $2.83\times10^{7}$ |
| Hole thermal velocity (cm/s) | $1.0\times10^{7}$ | $1.0\times10^{7}$ | $1.0\times10^{7}$ | $1.0\times10^{7}$ | $1.05\times10^{7}$ |

The current-voltage characteristics (J–V) and External Quantum Efficiency (EQE) vs. wavelength of the cell plots indicate an accurate agreement between the experimental data and the simulated one for two cases (i) with CdS as an electron transport layer, and (ii) with $In_2S_3$ as an electron transport layer. The results are summarized in fig.S1 and table S2.

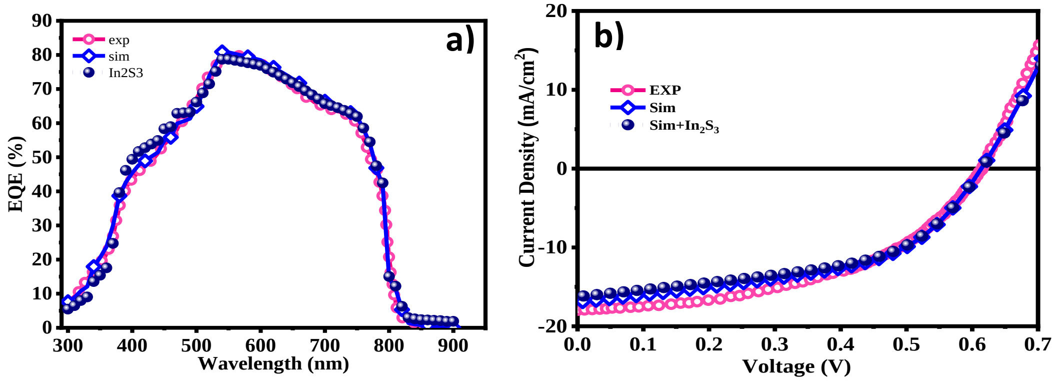


Figure S1: a) External Quantum Efficiency (EQE) vs. wavelength of the cell b) J–V characteristics for two cases (i) with CdS as a buffer layer, and (ii) with $In_2S_3$ as a buffer layer

Table S2: Functional parameters of experimental and simulated CBT (S, Se) solar cell

| Cell | Efficiency (%) | $V_{OC}$ (V) | $J_{SC}$ (mA/cm$^2$) | FF (%) |
|---|---|---|---|---|
| CBT (S, $Se_3$) - Experimental | 5.20 | 0.611 | 17.40 | 48.89 |
| CBT (S, Se) – Modeling (CdS) | 5.23 | 0.6141 | 16.93 | 50.29 |
| CBT (S, Se) – Modeling ($In_2S_3$) | 5.11 | 0.6151 | 16.24 | 51.18 |

The band structure of the cell and the total recombination are shown in fig.S2. As can be seen, $In_2S_3$ has a higher recombination rate in a small region of ~1 – 1.1 µm than the case of CdS, which leads to lower short-circuit current density ($J_{sc}$) (~0.7 mA). On the other hand, in the case of using $In_2S_3$, the cell records better open-circuit voltage ($V_{OC}$) due to the better band structure. In this case, the conduction electrons flow toward the front contact improves, and the energy levels gained better positions by utilizing the $In_2S_3$.

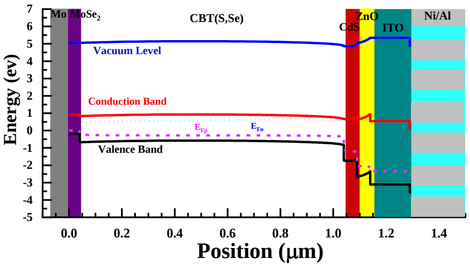


(a)

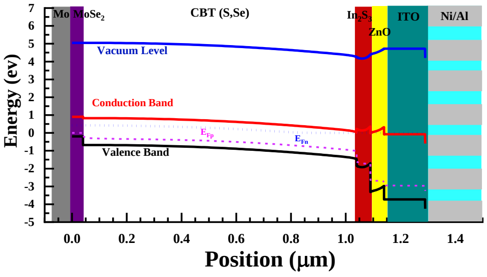


(b)

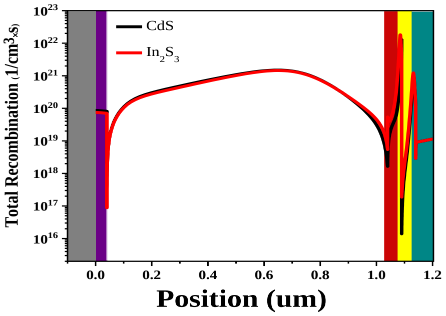


(c)

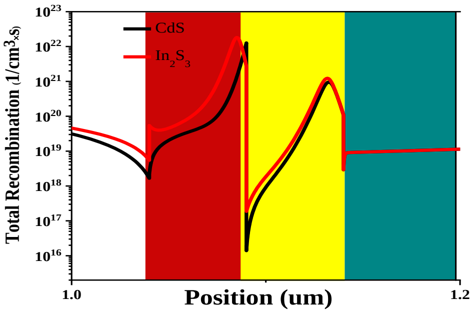


(d)

Figure S2: band structure of the cell in the case of a) CdS, and b) $In_2S_3$ as electron transport layers

c, d) total recombination of the cell in the case of CdS and $In_2S_3$ electron transport layers

## 2. References


[1] Y. Park, S. Lee, J. Yi, B.-D. Choi, D. Kim, J. Lee, *Thin Solid Films* **2013**, *546*, 337.

[2] S. M. Sze, K. K. Ng, *Physics of Semiconductor Devices*, John Wiley & Sons, **2006**.

[3] M. W. Rahman, S. I. Rahman, S. N. Ahmed, M. A. Hoque, in *2016 9th Int. Conf. Electr. Comput. Eng.*, **2016**, pp. 279–282.

[4] C. Persson, A. Zunger, *Phys. Rev. Lett.* **2003**, *91*, 266401.

[5] J. Dong, J. Shi, D. Li, Y. Luo, Q. Meng, *Appl. Phys. Lett.* **2015**, *107*, 73507.

[6] S. J. Fonash, in (Ed.: S.J.B.T.-S.C.D.P. (Second E. Fonash), Academic Press, Boston, **2010**, pp. 9–65.

[7] D. Shin, T. Zhu, X. Huang, O. Gunawan, V. Blum, D. B. Mitzi, *Adv. Mater.* **2017**, *29*, 1606945.

[8] D. Shin, B. Saparov, T. Zhu, W. P. Huhn, V. Blum, D. B. Mitzi, *Chem. Mater.* **2016**, *28*, 4771.

[9] D. Zhou, T. Zhou, Y. Tian, X. Zhu, Y. Tu, *J. Nanomater.* **2018**, *2018*.

[10] J. Ge, C. R. Grice, Y. Yan, *J. Mater. Chem. A* **2017**, *5*, 2920.

[11] J. Choi, S. Song, M. T. Hörantner, H. J. Snaith, T. Park, *ACS Nano* **2016**, *10*, 6029.

[12] M. Hashemi, M. Heidariramsheh, S. M. B. Ghorashi, N. Taghavinia, S. M. Mahdavi, *J. Photonics Energy* **2020**, *10*, 24001.
